# Vaccination completeness in children with rheumatic diseases: A longitudinal, observational multicenter cohort study in Switzerland

**DOI:** 10.3389/fped.2022.993811

**Published:** 2022-09-08

**Authors:** Tatjana Welzel, Jasmin Kuemmerle-Deschner, Constantin Sluka, Raffaella Carlomagno, Elvira Cannizzaro Schneider, Daniela Kaiser, Michael Hofer, Veronique Hentgen, Andreas Woerner

**Affiliations:** ^1^Pediatric Rheumatology, University Children’s Hospital Basel, University of Basel, Basel, Switzerland; ^2^Department of Pediatrics, Division of Pediatric Rheumatology, Autoinflammation Reference Center Tuebingen (arcT), University Hospital Tuebingen, Tuebingen, Germany; ^3^European Reference Network for Rare and Low Prevalence Complex Diseases, Network Immunodeficiency, Autoinflammatory and Autoimmune Diseases (ERN RITA), Tuebingen, Germany; ^4^Department of Clinical Research, University of Basel and University Hospital Basel, Basel, Switzerland; ^5^Pediatric Rheumatology, Centre Hospitalier Universitaire Vaudois, Lausanne, Switzerland; ^6^Pediatric Rheumatology, Hôpitaux Universitaires de Genève, Geneva, Switzerland; ^7^Pediatric Rheumatology, University Children’s Hospital Zurich, Zurich, Switzerland; ^8^Pediatric Rheumatology, Children’s Hospital Lucerne, Lucerne, Switzerland; ^9^Reference Center for Autoinflammatory Diseases CeRéMAIA, Versailles Hospital, Versailles, France

**Keywords:** vaccination adherence, infection risk, vaccination recommendations, immunosuppression, vaccination

## Abstract

**Introduction:**

Children with pediatric inflammatory rheumatic diseases (PRD) have an increased infection risk. Vaccinations are effective to avoid vaccine-preventable diseases. This study aimed to assess the vaccination completeness in Swiss PRD patients stratified by immunosuppressive treatment (IST).

**Materials and methods:**

This multicenter observational cohort study of PRD patients was performed in Basel, Geneva, Lucerne, Lausanne, and Zurich in PRD patients aged < 18 years included in the Juvenile Inflammatory Rheumatism Cohort. Completeness was assessed for i) the overall vaccination status (Swiss national immunization program (NIP) and specific additional PRD-recommended vaccinations), ii) for all and each vaccination of the NIP at PRD diagnosis and reference date (RefD) and iii) all and each specific additional PRD-recommended vaccination at RefD. Completeness was assessed over the disease course and stratified by IST.

**Results:**

Of 616 eligible patients, 234 children were analyzed. Of these, 147 (63%) were girls. Median age at PRD diagnosis was 6.5 years (IQR 2.9–10.3) and 10.9 years at RefD (6.9–14.3). The median follow-up since PRD diagnosis was 3 years (1.1–5.5). 120/234 children received IST. At RefD, overall vaccination completeness was 3.8% (9/234 children), completeness for the NIP vaccinations was 70.1% (164/234 children; IST 65%, no IST: 75.4%) and for all specific additional PRD-recommended vaccinations was 3.8% (9/234 children; IST 2.5%; no IST 5.3%). Vaccination completeness against pneumococcal disease, hepatitis B virus, and human papilloma virus (HPV) was 50.4, 20, 37.9%, respectively. In 25/35 children with negative varicella zoster virus history vaccination status was complete (IST: 94.4%, no IST: 47%). Annual non-live influenza vaccination was complete in 24.2% of children during IST; adherence decreased over the disease course.

**Discussion:**

This study identified a low overall vaccination completeness in children with PRD. Particularly, the completeness of specific additional PRD-recommended vaccinations was low. If not performed early after PRD diagnosis, vaccination status remained frequently incomplete. Close collaboration between pediatrician and rheumatologist to improve vaccination completeness is essential. Exchange of vaccination records, standardized assessment of specific PRD-recommended vaccinations and those of the NIP, and annual reminder for influenza vaccination are crucial to improve vaccination completeness in this vulnerable pediatric population.

## Introduction

Pediatric inflammatory rheumatic diseases (PRD) are associated with an increased risk of infections due to the underlying disease and the immunosuppressive treatment (IST), which is frequently needed to achieve remission (1, 2). The disease activity, the subtype of PRD, as well as the duration, dose and type of IST can influence the infection risk and the severity of infection-related complications (1–5). Several infections, which might be associated with high morbidity can be prevented by vaccinations (6, 7).

Countries all over the world summarize vaccination recommendations for the population against vaccine preventable diseases in their national immunization program (NIP). Many NIPs include recommendations for vaccinations against diphtheria, tetanus, pertussis, haemophilus influenzae type b, poliomyelitis, measles, mumps, rubella (MMR), and hepatitis b virus (HBV). Besides the NIP, several countries recommend specific additional vaccinations for patients with underlying chronic diseases and/or IST to protect them against vaccine preventable diseases. The European League against Rheumatism (EULAR) has proposed evidence-based vaccination guidelines for children with PRD (8), which were recently updated in collaboration with the Pediatric Rheumatology European Society (PRES) (9). In Switzerland, the Swiss Federal Office of Public Health (FOPH) has published specific vaccination recommendations in addition to the Swiss NIP for patients with IST and/or PRD (10).

There is evidence that inactivated vaccines are safe and effective in PRD patients regardless of IST, although immunogenicity might be reduced (11–13). Furthermore, live attenuated vaccinations can be administered safely in PRD patients without IST (8, 14). In the last years, studies on MMR and VZV vaccinations in children with PRD under IST reported that these vaccinations can be administered in special circumstances (15–18). However, a common approach was to postpone live attenuated vaccinations in PRD patients for a certain latency period after cessation of IST. With the recently published EULAR/PRES recommendations clinical practice may change for MMR booster and VZV vaccinations as those can be considered in patients with IST under specific circumstances (9). In order to reduce the risk from vaccine preventable infections in PRD, adherence to the NIP and to specific additional vaccination recommendations is crucial. However, vaccination status in PRD patients is frequently incomplete (19–21). As patients with rheumatic diseases are at increased risk of influenza, pneumococcal, herpes zoster and human papilloma virus (HPV) infections, adherence to vaccinations against those diseases is important (22). Nevertheless, it has been shown that the vaccination status against influenza and pneumococcal disease is frequently incomplete, even in patients on IST (23–26). A study in systemic lupus erythematosus patients revealed that influenza and pneumococcus vaccination status was complete in 45.2 and 32.2% of patients, respectively (27).

To date, no longitudinal data regarding adherence to the NIP and additional specific vaccination recommendations for children with PRD are available for Switzerland. Therefore, the aim of this study was to investigate the adherence to the Swiss NIP and to specific additional recommended vaccinations in children with PRD over the disease course stratified by IST.

## Materials and methods

### Study design

This longitudinal, multicenter observational cohort study of consecutive PRD patients was performed in Basel, Geneva, Lucerne, Lausanne, and Zurich to assess vaccination status in children and adolescents with PRD. Patients aged < 18 years with a signed informed consent for the Juvenile Inflammatory Rheumatism Cohort (JIR cohort), a PRD diagnosis and available data for date of diagnosis, age and gender were eligible for this study. Of these, all patients with an available vaccination record at last follow-up visit were included. Children with positive maternal history for hepatitis B at birth or prematurity were excluded. In addition, all children with last follow-up visit more than 12 months before the reference date were excluded. The reference date was defined as date of data extraction (12th April 2016). The patients‘ data were captured in the JIR cohort database. The JIR cohort is an international multicenter data registry for the long-term follow-up of patients with rheumatic inflammatory diseases [NCT02377245, PB_2016-00868 (2017/13)]. Captured data was provided as a coded data extract by the JIR data manager after an approval from the JIR data access committee was obtained. The vaccination status according the Swiss NIP was assessed at date of PRD diagnosis (baseline visit) and at reference date. Vaccination status of specific additional vaccinations recommended in patients with PRD was assessed at reference date. In addition, vaccination status was assessed over the disease course, censored at 72 months after PRD diagnosis for the vaccination status according to the NIP and specific additional recommended PRD vaccination. Data was stratified by IST. An approval from the responsible ethic committee was obtained (2022-00752).

### Characteristics of the study cohort

The characteristics included gender, age, date of last follow-up visit and PRD diagnosis. Diagnoses were subcategorized into JIA, connective tissue diseases (CTD), uveitis, vasculitis, and autoinflammatory diseases (AID). In addition, treatment was assessed over the disease course. Treatment was categorized as non-IST and IST according to the Swiss recommendations (10). Treatment with (i) methotrexate (MTX) dosed 20 mg/week or 0.4 mg/kg/week, (ii) and other conventional disease modifying antirheumatic drugs (cDMARDs) as well as (iii) biological disease modifying antirheumatic drugs (bDMARDs) such as tumor-necrosis-factor (TNF) inhibitors, abatacept, interleukin (IL)-1 inhibitors, IL-6 inhibitors, rituximab, and (iv) systemic corticosteroids with prednisone/prednisone–equivalent doses of ≥ 0.5 mg/kg/day (or ≥ 20 mg/day absolute) for ≥ 14 days were defined as IST (10).

### Swiss national vaccination schedule and specific additional recommended vaccinations

The 2016 Swiss NIP included vaccinations against diphtheria, tetanus, pertussis, poliomyelitis, haemophilus influenza type B, HBV and MMR (Table 1) (28). In addition, HPV vaccination was recommended in healthy girls aged 11–14 years with a two dose scheme (Table 1) (28). In Switzerland, the vaccination against HBV can be performed voluntarily in infancy, but should be performed between 11 and up to 16 years. In addition, VZV vaccination is recommended in Switzerland for adolescents aged 11–15 years with negative VZV history or those who are still seronegative at that age (28). Vaccination against pneumococcal disease is not recommended routinely for healthy children, but can be performed voluntarily (28). With diagnosis of a PRD, the specific additional recommended vaccinations, in the following described as PRD indication vaccination, should be performed as soon as possible (10). The PRD indication vaccinations include vaccinations against pneumococcal disease (if not done voluntarily before PRD diagnosis), HBV (if not done voluntarily before PDR diagnosis) and VZV (if history is negative). Furthermore, the non-live influenza vaccination should be performed annually in children with immunosuppression (10, 28). In children with IST younger than 9 years the non-live influenza vaccination should be performed in the first year of vaccination two times with an interval of 1 month between each vaccination, followed by one vaccination annually as long as IST is administered (28). In addition, HPV vaccination should include three doses in girls with PRD aged 11–14 years (28).

**TABLE 1:** Swiss national vaccination schedule (28).

Age	Vaccination against						
	DTP	Poliomyelitis	Hib	HBV	MMR	VZV	HPV
2 Months	**x**	**x**	**x**	**(x)**			
4 Months	**x**	**x**	**x**	**(x)**			
6 Months	**x**	**x**	**x**	**(x)**			
12 Months					**x**		
15–24 Months	**x**	**x**	**x**	**(x)**	**x**		
4–7 Years	**x**	**x**					
11–14/15 Years	**x**			**x**		**x^1^**	**♀: x**

DTP, diphtheria, tetanus, pertussis; Hib, Haemophilus influenza virus b; HBV, Hepatitis virus B; MMR, Mumps, measles, rubella; VZV, Varicella zoster virus; HPV, Human papilloma virus; () voluntary; **♀** girls, ^1^ If VZV history negative/seronegative.

### Definition and assessment of completeness

Overall vaccination completeness was defined as a combination of a complete vaccination status for all vaccinations (i) recommended in the Swiss NIP and (ii) all PRD indication vaccinations. For a complete vaccination status according to the Swiss NIP, all recommended vaccinations (age dependent) had to be complete. If vaccinations were recommended during age ranges the upper age limit was used. In children at ages less than 2 years of life, a delay of a maximum of 1 month was allowed, at age ≥ 2 years a maximum delay of 3 months was allowed. For a complete PRD indication vaccination status, all recommended vaccinations had to be complete respecting latency periods recommended for live attenuated vaccinations (10) (Supplementary Table 1). For vaccinations of special interest (non-live influenza and pneumococcal disease), the vaccination status was defined as complete if all age-dependent recommended vaccinations were performed. The vaccination completeness against pneumococcal diseases had to fulfill the age dependent recommendations (age 2–6 months: 4 doses, age 7–11 months: 3 doses, age 12–23 months: 2 doses, age > 24 months: 1 doses). For influenza, vaccination status was defined as complete in all PRD patients without IST. In those with IST influenza vaccination status was defined as complete, if annual non-live influenza vaccinations during IST between October and March were performed with two vaccinations in the first year if patients were aged younger than 9 years. If VZV history was positive in PRD patients the vaccination status was defined as complete. In case of negative VZV history or if patient was seronegative at PRD diagnosis, the vaccination against VZV had to be performed in line with recommendations in respect to IST (10). If the patient received IST immediately with diagnosis or before 9 months after PRD diagnosis the VZV vaccination status was considered as complete until IST was censored and latency period according to the type of IST was over (10).

The overall vaccination completeness (NIP and PRD indication vaccinations), the completeness of all PRD indication vaccinations and each specific PRD indication vaccination was assessed at reference date. In addition, the overall vaccination completeness (NIP and PRD indication vaccinations), the completeness of all specific PRD indication vaccinations and the completeness of vaccinations of specific interest (influenza, pneumococcal disease) were assessed over the disease course. The vaccination completeness for vaccinations recommended by the NIP was assessed at PRD diagnosis (baseline visit) and reference date as well as over the disease course.

### Outcome

Primary outcome was defined as overall completeness of the vaccination status for the whole cohort of children with PRD stratified by IST at reference date and over the disease course.

Secondary outcomes variables included: (1) Complete vaccination status according to the NIP for the whole cohort at PRD diagnosis (baseline visit) and reference date and stratified by IST at reference data and over the disease course. (2) Complete PRD indication vaccination status according to the specific additional PRD vaccination recommendations for the whole cohort and stratified by IST at reference date and over the disease course. (3) Complete vaccination status against (i) VZV, (ii) influenza, (iii) pneumococcal disease, (iv) HBV and (v) HPV for the whole cohort and stratified by IST at reference date and for those of specific interest (influenza, pneumococcal disease) over the disease course.

### Analysis

The demographics were analyzed using descriptive statistics. Mean, median, standard deviation and interquartile range (IQR) were used for continuous variables, absolute and relative frequencies were used for categorial variables. The FOPH vaccination recommendations were transferred into decision trees in R code using the visual language editor Drakon (version 1.29). All statistical analyses were conducted with R (version 4.2.0).

## Results

### Characteristics of the study cohort

**FIGURE 1:**
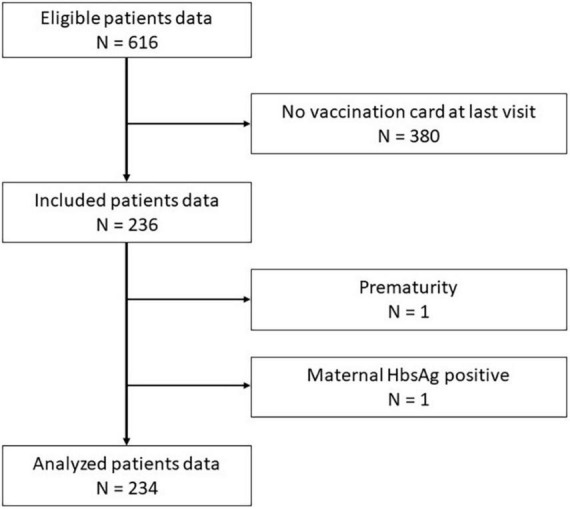
Flow chart study cohort. *HbsAg*, Hepatitis B antigen.

A total of 616 patients were eligible. Of these, 236 PRD patients (38%) had an available vaccination record at last follow-up visit and were included in this study. The follow-up visit furthest from the reference date included in the study was dated the 27th April 2015. The majority (75%) of all patients had their follow-up visits in 2016 (IQR 01/2016–03/2016). Two patients had to be excluded; one due to prematurity and one due to positive maternal HBV antigen (Figure 1). Data analysis was performed in a total of 234 children with PRD. Of these, 147 (63%) were girls. PRD diagnosis were distributed accordingly: JIA was diagnosed in 162 children (69.2%). Of these, 14 patients had a systemic JIA and 25 children had a concomitant uveitis. An isolated idiopathic uveitis was diagnosed in 3 children (1.3%). An AID was diagnosed in 51 children (21.8%), 8 children (3.4%) had a vasculitis and 10 children (4.3%) were diagnosed with a CTD. Median age at PRD diagnosis was 6.5 years (IQR 2.9–10.3). At PRD diagnosis, 9 patients were aged ≥ 15 years (four girls, five boys). Median age at reference date was 10.9 years (IQR 6.9–14.3). The median follow-up time for the whole cohort was 3 years (IQR 1.1–5.5) since date of diagnosis. Those children with PRD who received IST had a longer follow-up time (3.6 years; IQR 2.1–6.94) compared to those who never received IST. A total of 120/234 children (51.3%) received an IST. Of these, 87/120 children (72.5%) still received IST at reference date. During the observation time 81 children have ever received bDMARDs, 77 children received cDMARDs and 14 patient systemic steroids (Table 2). In this cohort children who were treated with bDMARDs received abatacept, adalimumab, anakinra, canakinumab, etanercept, golimumab, infliximab, or tocilizumab.

**TABLE 2:** Characteristics of the study cohort.

Characteristic	PRD data set analyzed
**General characteristics**	
Median age at PRD diagnosis, years (IQR)	6.5 (2.9–10.3)
Girls (%)	147 (63)
Girls ≥ 15 years at baseline visit (%)	4 (2.7)
Girls ≥ 15 years at last visit (%)	29 (19.7)
**Distribution of PRD diagnosis, patients (%)**	
Autoinflammatory diseases	51 (21.8)
Connective tissue diseases	10 (4.3)
Juvenile idiopathic arthritis^a^	162 (69.2)
Idiopathic uveitis	3 (1.3)
Vasculitis	8 (3.4)
**Immunosuppressive treatment^b^**	
cDMARD	77
bDMARD	81
Systemic corticosteroids	14

IQR, Interquartile range; PRD, Pediatric rheumatic disease; cDMARD, conventional disease modifying antirheumatic drugs; bDMARD, biological disease modifying antirheumatic drugs.

^a^Includes children with concomitant uveitis and children with systemic juvenile idiopathic arthritis.

^b^Patients may obtain multiple drugs of one drug group during the observation period.

### Outcome

#### Primary outcome

**FIGURE 2:**
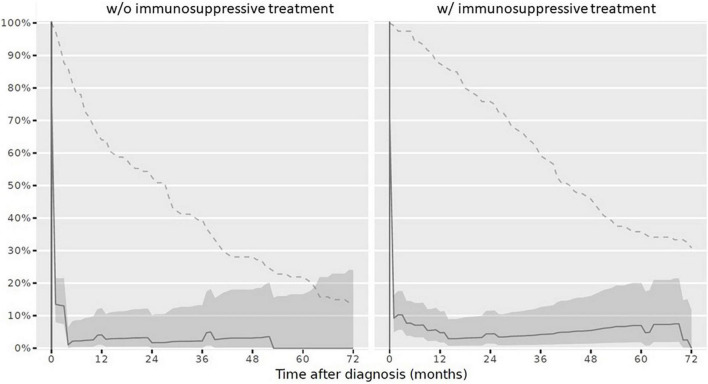
Complete overall vaccination status in children after PRD diagnosis according to the Swiss national vaccination schedule and specific additional PRD vaccination recommendations. Legend: *y-axis* Completeness (% patients vaccinated completely, solid line with 95% confidence interval), *x-axis* Sample size (% patients available, dashed line). *PRD*, pediatric patients with rheumatic inflammatory diseases. *w/o*, Without immunosuppressive treatment *w/*with immunosuppressive treatment.

At reference date, the overall vaccination status was complete in 9/234 children (3.8%). Stratified by IST, 3/120 children (2.5%) with IST had a complete vaccination status and 6/114 children (5.3%) never receiving IST had a complete vaccination status. The overall vaccination status after PRD diagnosis over the disease course decreased (Figure 2).

#### Secondary outcomes

**FIGURE 3:**
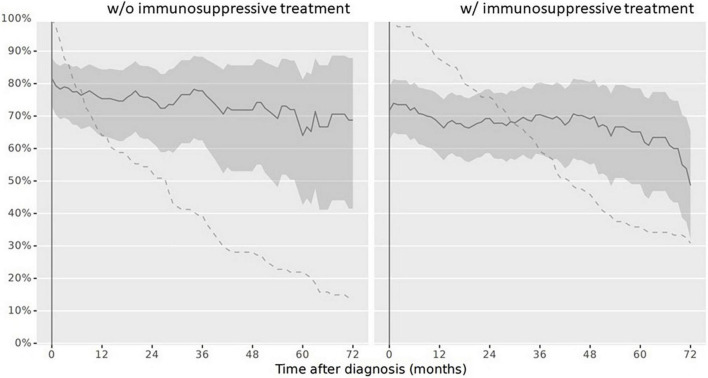
Complete vaccination status according to the Swiss national vaccination schedule in children after PRD diagnosis. Legend: *y-axis* Completeness (% patients vaccinated completely, solid line with 95% confidence interval), *x-axis* Sample size (% patients available, dashed line). Abbreviation: *PRD* pediatric patients with rheumatic inflammatory diseases. *w/o*, Without immunosuppressive treatment *w/* with immunosuppressive treatment.

**FIGURE 4:**
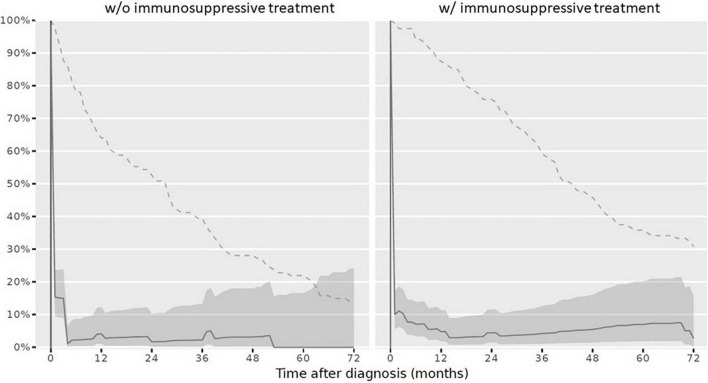
Complete vaccination status according to the specific additional vaccination recommendations in children after PRD diagnosis. Legend: *y-axis* Completeness (% patients vaccinated completely, solid line with 95% confidence interval); *x-axis* Sample size (% patients available, dashed line). *PRD* pediatric patients with rheumatic disease. *w/o*, without immunosuppressive treatment *w/* with immunosuppressive treatment.

**FIGURE 5:**
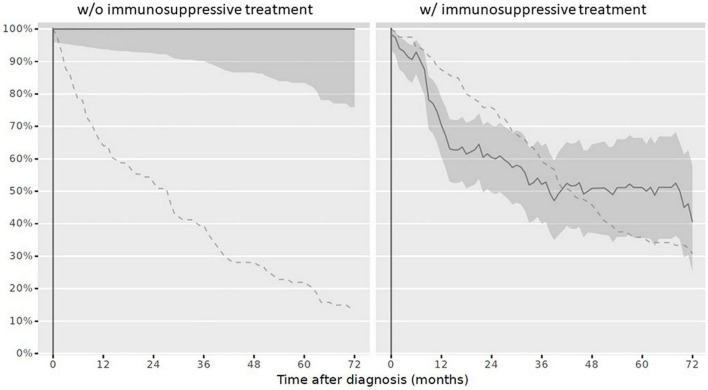
Complete vaccination status against influenza in children after PRD diagnosis. Legend: *y-axis* Completeness (% patients vaccinated completely, solid line with 95% confidence interval); *x-axis* Sample size (% patients available, dashed line). *PRD*, pediatric patients with rheumatic disease. *w/o*, without immunosuppressive treatment, *w/*with immunosuppressive treatment.

**FIGURE 6:**
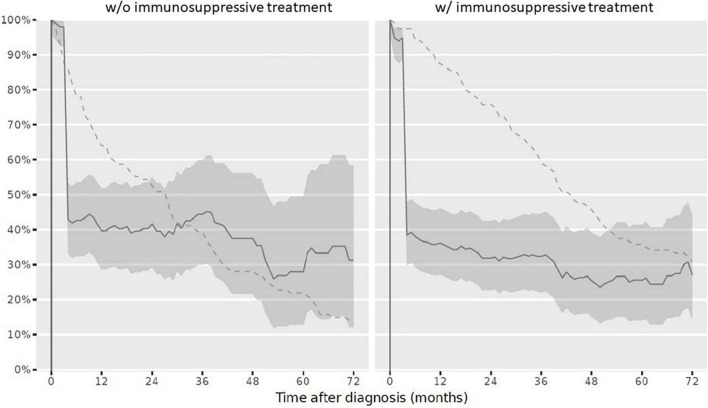
Complete vaccination status against pneumococcal diseases in children after PRD diagnosis. Legend: *y-axis* Completeness (% patients vaccinated completely, solid line with 95% confidence interval); *x-axis* Sample size (% patients available, dashed line). *PRD* pediatric patients with rheumatic disease. *w/o*, without immunosuppressive treatment, *w/*with immunosuppressive treatment.

(1) At diagnosis, 179/234 children (76.5%) had a complete vaccination status according to the NIP. At reference date, 164/234 children (70.1%) had a complete vaccination status according to the NIP. When stratifying the cohort for vaccination completeness by IST at reference date, children with IST more often had an incomplete vaccination status. The vaccination status in line with the NIP was complete in 86/114 children (75.4%) never receiving an IST and in 78/120 children (65%) with IST at reference date. Over the disease course, particularly vaccinations against diphtheria, tetanus, and pertussis were missed. Vaccination status for live attenuated vaccinations (mumps, measles and rubella) improved and was more often complete in children with IST compared to those without at reference date (Table 3). Completeness of the vaccination status decreased in those with and without immunosuppression over the disease course after PRD diagnosis was made (Figure 3).
(2) Complete vaccination status for all specific PRD indication vaccinations was assessed in 9/234 children (3.8%) at reference date. Of these, three children received IST and 6 children had never received IST. When this data was compared to the overall vaccination completeness, the low completeness resulted from an incomplete specific PRD indication vaccination status. Over the disease course the vaccination status remained incomplete in the majority of patients (Figure 4).
(3) For each specific recommended PRD indication vaccination the completeness was assessed accordingly:
  (i) *VZV*: A VZV history was documented for 134/234 children (57.3%). Of these, the majority was seropositive due to wild type infection or due to voluntarily vaccination before PRD diagnosis (*n* = 99). Of the remaining 35 children with negative VZV history, 17 children never received IST during the observation period. At reference date, 25 children (71.4%) with negative VZV history had a complete vaccination status. Of these, 17 children received IST (Table 4).
  (ii) *Influenza*: At reference date, 29/120 children (24.2%) with IST were annually vaccinated between October and March during IST. Over the disease course censored at 72 months, the vaccination adherence to the annually recommended non-live influenza vaccination decreased (Figure 5).
  (iii) *Pneumococcal disease*: At reference date, 118/234 children (50.4%) had received vaccination against pneumococcal disease according to age and in line with the specific additional PRD recommendations. Half of patients, who had a complete vaccination status received IST (59/120 children; 49.2%), the other half never received IST (59/114 children; 51.8%). If vaccination was not performed early after diagnosis, vaccinations status remained more often incomplete over the disease course (Figure 6).
  (iv) *HBV*: Complete vaccination status against HBV was documented in 47/234 children (20%) at reference date. Of these, 23/120 children (19.2%) with IST were vaccinated complete.
  (v) *HPV*: In total, 29/167 girls were aged 15 years and older at last visit and should have received HPV vaccinations before reference date. HPV vaccination status was complete in 11/29 girls with PRD (37.9%). Stratified by IST, 5/16 girls (31.3%) with IST had a complete HPV vaccination status compared to 6/13 girls (46.2%) never receiving IST.

**TABLE 3:** Vaccination status in children with PRD according to the Swiss national vaccination schedule.

Vaccination against	Diagnosis date	Reference date		
	Whole cohort (%)	Whole cohort (%)		
Diphtheria	213 (91)	199 (85)		
Tetanus	213 (91)	199 (85)		
Pertussis	208 (88.9)	185 (79.1)		
H. influenza type B	213 (91)	211 (90.2)		
Poliomyelitis	206 (88)	199 (85)		
		Children with IST (%)		Children without IST (%)
Measles	208 (88.9)	113/120 (94.2)		104/114 (91.2)
Mumps	206 (88)	113/120 (94.2)		103/114 (90.4)
Rubella	206 (88)	113/120 (94.2)		103/114 (90.4)

PRD, Pediatric rheumatic disease; IST, Immunosuppressive treatment.

**TABLE 4:** VZV history and vaccination status in children with PRD at reference date.

VZV history	VZV vaccination status	Children with IST (%)	Children without IST (%)
Negative	Complete	17/18 (94.4)	8/17 (47.1)
	Incomplete	1/18 (5.6)	9/17 (52.9)
Positive	Complete^a^	58/120 (48.3)	41/114 (36)
Unknown	n.a.	44/120 (36.7)	56/114 (49)

PRD, Pediatric rheumatic inflammatory diseases; IST, Immunosuppressive treatment; n.a., not applicable.

^a^Children with positive history were defined as complete as no vaccination was anymore needed.

## Discussion

This is the first longitudinal cohort study analyzing the vaccination status in Swiss children with PRD. The completeness of the overall vaccination status in children with PRD in this study was only 3.8% (9/234 children). Children with PRD never receiving IST had a more complete vaccination status compared to those receiving IST. Although vaccination status against mumps, measles and rubella increased over the disease course, the completeness of the vaccination status including all recommended vaccinations in the NIP decreased over the disease course (at PRD diagnosis: 76.5%, reference date: 70.1%) mainly due to missed inactivated vaccinations. However, the low overall vaccination status resulted mainly from non-adherence to specific additional PRD indication vaccination recommendations. Particularly, vaccination completeness against HBV (20%) and non-live influenza was low (24.2%). Influenza vaccination adherence decreased over the disease course.

In this PRD cohort the completeness of the NIP vaccination status decreased over the disease course. Nearly every third child with PRD (30%) had an incomplete vaccination status at reference date. The availability of the vaccination record at the rheumatologist visit was low.

Diallo et al. studied the vaccination status in children with chronic diseases (29). Of 207 identified children with chronic diseases, vaccination data was available in 146 patients (71%). Of these, 47% complied with the NIP (29). Morin et al. studied vaccination completeness in children diagnosed with JIA at age of 2.5, 10.5 years and at last clinical visit (20). Of all children with JIA, who had received a reminder to bring their vaccination record to the scheduled rheumatologist appointment, vaccination records were not available for 93 patients. Although Morin et al. assessed high completeness (up to 99%) to some vaccinations, completeness for all vaccinations together recommended in the NIP was low (20). A complete vaccination status was assessed in 61% of JIA patients at their last clinical rheumatology appointment (20). Minden et al. analyzed vaccination records of 715 JIA patients captured in the German Kinderkerndokumentation (19). Data indicated that every third JIA patient had an incomplete vaccination status. The incomplete vaccination status mainly resulted from non-adherence to booster vaccinations (19). Whereas good to very good vaccination adherence to the NIP was assessed in children with JIA until school age, tetanus and diphtheria booster vaccination were more likely missed in JIA patients aged 7–11 and 12–17 years compared to healthy controls (19). For French children with fever syndromes the vaccination status according to the NIP was complete in 32% of patients aged 2 years, in 28% aged 7 years, in 6% at age of 15 years and in 44% of children at last outpatient visit (30). Bizjak et al. reported vaccination data for 187 children with PRD (21). Children aged 6 years or older at time of PRD diagnosis were 2.75 (1.47–5.12) times more likely to be in line with the recommended NIP compared to patients, who were diagnosed younger (21). In this study the decrease in completeness for vaccinations recommended in the NIP resulted mainly from missed inactivated vaccinations over the disease course. In addition, vaccination status was more often incomplete in those children receiving IST. This is in line with Bizjak et al. (21), who assessed in 84.2% of children with PRD never receiving IST a complete vaccination status, whereas only 64.7% treated with non-bDMARD and 45.9% treated with bDMARD at any time had a complete vaccination status (21). For children never receiving IST the vaccination status was 2.97 times (1.2–7.07) more likely to be up to date compared to those receiving non-bDMARD treatment at any time and 6.24 times (2.58–15.33) more likely compared to patients receiving bDMARD treatment at any time (21). In a cross-sectional anonymous survey study, 50% of pediatricians stated that they are hesitant to adhere to the NIP in PRD patients without specialist input (31). Main reasons for this were fear to provoke a disease flare (43%) and inability to deal with parental concerns (54%) (31). This uncertainty might increase if a child with PRD receives IST, highlighting the importance of the new recommendations of EULAR/PRES, that the treating rheumatologists should assess yearly the vaccination status of their PRD patients (9). This is particularly of relevance as with the PRD diagnosis the child might have a closer contact to the rheumatologists as to the pediatrician, who is usually responsible for the primary care including vaccinations. In line with this, the availability of the vaccination records during the rheumatology appointment has to be improved. The parents and patients have to be sensitized to bring the vaccination records to the specialist appointment. In addition, transfer of the vaccination record together with the patients‘ referral from the pediatrician to the rheumatologist may increase availability. The rheumatologist should transfer information to the pediatrician when and which vaccinations should be performed in children with PRD. Regular assessment of the vaccination record and close collaboration between the pediatric rheumatologist and the pediatrician/primary care provider will improve completeness of the vaccination status and adherence to vaccination recommendations.

In this study, particularly the adherence to the specific additional recommended PRD indication vaccinations was very low and was mainly responsible for the assessed low overall vaccination completeness. As vaccination completeness for the specific PRD indication vaccinations decreased over the disease course, it seems that if children are not vaccinated early after PRD diagnosis the vaccination status remained incomplete.

For children with PRD as well as for adults with rheumatic diseases, specific additional vaccination recommendations are available (8–10, 14, 32). The specific additional vaccinations aim to protect these vulnerable patients against vaccine preventable diseases, as several of those might have a more severe disease course with higher risk for disease complications (22, 33–36). However, vaccination adherence to these specific additional indication vaccinations, including vaccinations against pneumococcal disease and influenza, seem to be low. Diallo et al. found that 13/118 children with chronic diseases (11%) had completely received their specific additional recommended vaccinations (29). Rollet-Cohen studied 14 children with AID and IST and none adhered to the specific additional recommended indication vaccinations against VZV, influenza or pneumococcal disease (30). Bizjak et al. assessed at least one vaccination against VZV in 25/187 children with PRD (13.4%), in 8/187 children (4.3%) against pneumococcal disease and in 19/187 children (10.2%) against influenza at last clinical visit (21). In this study, the vaccination status for specific recommended vaccinations was low, but appears higher compared to previously studies. The vaccination status was complete against pneumococcal diseases in 50.4% of children with PRD, against VZV -if VZV history was negative- in 71.4% of children with PRD and the annually non-live influenza vaccination during IST was performed in 24.2% of children at reference date. However, adherence to the annually non-live influenza vaccination during IST was lower and decreased over the disease course in this study. In contrast Alauzet et al. who reported a stable adherence with 42–43% to the annual influenza vaccination over a 4-year period for children with chronic diseases (37). There is data that patients who receive a specific recommendation for influenza vaccination by their physicians are significantly more likely to be vaccinated compared to those patients who did not receive such a recommendation (57% vs. 15%) (38). Recently, 1,015 adult patients with rheumatic diseases were telephone interviewed regarding their adherence to influenza vaccination recommendation in the years 2019/2020 and 2020/2021 (39). The main reasons not being influenza vaccinated were the absence of a specific recommendation from the treating physicians, as well as the belief that vaccinations would not be helpful, fear of potential side-effects, and other reasons including forgetfulness (39). The strong association between being influenza vaccinated and having received a recommendation for the annual influenza vaccination was also demonstrated in childhood (40, 41). Moreover, the parental behavior impacts the likelihood of being vaccinated against influenza, as the influenza vaccination of the child is strongly associated with the parents‘ annual influenza vaccination status (40). Data of this study have shown, that adherence to the non-live annual influenza vaccination during IST decreases over the disease course, therefore it has to be emphasized that regular annual reminder by the treating physician (written or orally) are crucial. In addition, vaccination status for specific additional PRD indication vaccination remained often incomplete if they were not performed early after diagnosis. The recommended yearly vaccination record assessment by the rheumatologist (9) will help to identify specific additional PRD indication vaccinations missed at PRD diagnosis and will therefore improve completeness of the specific indication vaccinations over the disease course.

This study has several limitations. Strict inclusion and exclusion criteria resulted in a small sample size. Although only data of children with follow-up visits not older than 12 months were analyzed, in some children, particularly in those aged < 1 year vaccinations might have been missed at reference date. However, vaccination completeness seems to be comparable or higher compared with previous studies. Data assessed in this study result from a prospective real-life cohort and the standardized data capture and clear definitions resulted in high quality data. Unfortunately, reasons why vaccination status is incomplete could not be assessed as this information is not captured in the cohort. Therefore, it remains unclear if the physician has missed to perform the vaccinations or if the parents/patients have refused the vaccinations. Moreover, as disease activity was not implemented in the analysis, it is possible that vaccinations might be postponed due to disease activity. However, one strength of this study is the stratification by IST and that vaccination status was analyzed over the disease course censored at 72 months, taking into account the individual follow-up time of the cohort.

## Conclusion

In conclusion, this study indicates that the overall vaccination completeness is low, and that children with PRD and IST are often more incompletely vaccinated compared to those never receiving IST. The incompleteness seems to result from (i) missed inactivated vaccinations recommended in the NIP and (ii) incomplete specific additional PRD indication vaccinations status. In this study the specific additional PRD vaccination status remained more likely incomplete, if vaccinations were not performed early after diagnosis and adherence to the annual non-live influenza vaccination decreased over the disease course. Therefore, close collaboration between the primary care provider/pediatrician and the pediatric rheumatologist is important to improve the adherence to vaccination recommendations. This may include (i) transfer of the vaccination record together with the patients‘ referral to the pediatric rheumatologist by the pediatrician/primary care provider, (ii) information of specific additional vaccinations recommendation by the rheumatologist to the patient/its family and the pediatrician/primary care provider, (iii) standardized and annual regular assessment of the vaccination status e.g., with checklists by the pediatric rheumatologist and the primary care provider/pediatrician, as well as (iv) regular annually reminder for the non-live influenza vaccination during IST. These measures might improve the completeness of the vaccination status in children with PRD and will help to avoid infections with vaccine preventable diseases in this vulnerable pediatric population.

## Data availability statement

The data analyzed in this study has been captured in the JIR cohort. Requests to access the dataset should be directed to Francois Hofer, info@jircohorte.ch.

## Ethics statement

The studies involving human participants were reviewed and approved by the Ethikkommission Nordwest-und Zentralschweiz. Written informed consent to participate in JIR cohort was provided by the participants’ legal guardian/next of kin.

## Author contributions

TW, JK-D, CS, RC, EC, DK, MH, VH, and AW contributed to the study design and conceptualization. TW, EC, DK, RC, MH, and AW involved in data curation and data gathering. TW, CS, and AW mainly involved in data analyzing and prepared the original draft. JK-D, RC, EC, DK, MH, and VH reviewed and edited. All authors have approved this version and agreed to the submission of this manuscript.
