# Supplementary material for: Vaccination completeness in children with rheumatic diseases: A longitudinal, observational multicenter cohort study in Switzerland
Source: Front Pediatr. 2022 Sep 8;10:993811. doi: 10.3389/fped.2022.993811 (PMC9493270; doi:10.3389/fped.2022.993811)
Supplement: Supplementary file 1 [file Table_1.DOCX]

**Supplementary material**

Supplementary Table 1: Recommended latency periods between stop of immunosuppressive treatment and administration of live attenuated vaccinations [1]

| **Treatment** | **Latency period (minimum)** |
| --- | --- |
| Corticosteroids  (< 2 weeks, prednisone equivalent: <0.5 mg/kg/day or < 20 mg/day) | Not needed |
| Sulfasalazine  Hydroxychloroquine |  |
| Corticosteroids  (≥ 2 weeks, prednisone equivalent: ≥0.5 mg/kg/day or ≥ 20 mg/day) | 1 month |
| Azathioprine  6-Mercaptopurine  Cyclosporine A  Mycophenolate  Cyclophosphamide  Tacrolimus | 3 months |
| Methotrexate (> 0.4mg/kg/week or >20mg/week) | 3 months |
| Leflunomide | 2 years |
| Etanercept | 1 to 3 months |
| Rituximab | 12 months |
| Infliximab  Adalimumab  Golimumab  Certolizumab  Abatacept  Tocilizumab  Ustekinumab | 3 months |

Abbreviation: *mg* milligram, *kg* kilogram,

Reference:

1. *Bundesamt für Gesundheit (BAG) und Eidgenössische Kommission für Impffragen (EKIF): Impfprinzipien und Empfehlungen für Personen mit autoimmun-entzündlichen rheumatischen Erkrankungen.* Buelletin 2014. **8**: p. 159 - 161.
